# Non-specific LTD at parallel fibre - Purkinje cell synapses in cerebellar cortex provides robustness against local spatial noise during pattern recognition

**DOI:** 10.1186/1471-2202-12-S1-P314

**Published:** 2011-07-18

**Authors:** Karen Safaryan, Reinoud Maex, Rod Adams, Neil Davey, Volker Steuber

**Affiliations:** 1 Science and Technology Research Institute, University of Hertfordshire, Hatfield, Herts, AL10 9AB, UK

## 

Coincident parallel fibre (PF) and climbing fibre (CF) input to cerebellar Purkinje cells (PCs) induces long-term depression (LTD) of PF synapses. PF LTD is widely considered as a synaptic mechanism of cerebellar learning and pattern recognition [1,2]]. It has traditionally been assumed that PF LTD is specific to the synapses that receive PF input when a CF signal arrives at the PC. However, more recent experiments have shown that LTD takes place not only at the activated PF synapses, but also at those nearby [3]. The amount of LTD decreases as a Gaussian function of distance from the activated PF synapses [3]. The functional role of this non-specific LTD is not clear.

We have previously studied the effect of non-specific synaptic plasticity (NSSP) on pattern recognition in an artificial neural network. In the absence of noise in the input patterns, introducing NSSP resulted in a decreased pattern recognition performance, similar to the loss of performance reported for unsupervised learning based on Oja’s rule [4]. However, in the presence of spatial noise in the input patterns, NSSP improved the pattern recognition performance [5]. These results were confirmed using a PC model with a simplified morphology comprising 1474 spines and 100 PF synapses / spine [5].

The present study investigates the effect of non-specific LTD on pattern recognition in a PC model with a realistic distribution of PF synapses and one synapse / spine. We placed 14,740 spines along the thin dendrites and distributed them uniformly, but with random orientation. Following the experimental evidence we modeled non-specific LTD as a Gaussian function of distance. We presented stored (noisy) and novel patterns to the PC model and determined the pattern recognition performance by measuring signal-to-noise (s/n) ratios for the difference in pauses in PC spiking caused by the presentations of input patterns; as shown previously [2], pauses in spiking were the best criterion for the PC to distinguish between stored and novel patterns.

In the absence of local spatial noise the pattern recognition performance deteriorated in PC models with non-specific LTD. For non-specific LTD with standard deviations of ≥ 1.25µm, the s/n ratio approached zero and pattern recognition became impossible.

Introducing local spatial noise in the patterns also decreased the pattern recognition performance, both in the presence and absence of non-specific LTD. However, when the amount of noise in the stored patterns was ≥ 30%, PC models with non-specific LTD outperformed models where LTD was specific to active PF synapses. This improvement of pattern recognition by non-specific LTD was particularly pronounced when the spatial distribution of noise in the input patterns matched the spatial spread of the non-specific synaptic plasticity. In simulations where the extent of the spread of the noise exceeded the spread of the non-specific LTD, the beneficial effect of the non-specific plasticity disappeared in a graded manner with an increasing spread of noise.

Our simulation results predict that a possible computational function for non-specific LTD at PF – PC cell synapses is to provide robustness against local spatial noise.
